# Emerging Functions of Nanostructured Porous Silicon—With a Focus on the Emissive Properties of Photons, Electrons, and Ultrasound

**DOI:** 10.3389/fchem.2019.00273

**Published:** 2019-04-24

**Authors:** Nobuyoshi Koshida, Toshihiro Nakamura

**Affiliations:** ^1^Graduate School of Engineering, Tokyo University of Agriculture and Technology, Fuchu, Japan; ^2^Department of Electrical and Electronic Engineering, Hosei University, Tokyo, Japan

**Keywords:** porous silicon, nanocrystal, colloidal silicon, photoluminescence, ballistic electron emission, thermoacoustic

## Abstract

Recent topics of application studies on porous silicon (PS) are reviewed here with a focus on the emissive properties of visible light, quasiballistic hot electrons, and acoustic wave. By exposing PS in solvents to pulse laser, size-controlled nc-Si dot colloids can be formed through fragmentation of the PS layer with a considerably higher yield than the conventional techniques such as laser ablation of bulk silicon and sol-gel precursor process. Fabricated colloidal samples show strong visible photoluminescence (~40% in quantum efficiency in the red band). This provides an energy- and cost-effective route for production of nc-Si quantum dots. A multiple-tunneling transport mode through nc-Si dot chain induces efficient quasiballistic hot electron emission from an nc-Si diode. Both the efficiency and the output electron energy dispersion are remarkably improved by using monolayer graphene as a surface electrode. Being a relatively low operating voltage device compatible with silicon planar fabrication process, the emitter is applicable to mask-less parallel lithography under an active matrix drive. It has been demonstrated that the integrated 100 × 100 emitter array is useful for multibeam lithography and that the selected emission pattern is delineated with little distortion. Highly reducing activity of emitted electrons is applicable to liquid-phase thin film deposition of metals (Cu) and semiconductors (Si, Ge, and SiGe). Due to an extremely low thermal conductivity and volumetric heat capacity of nc-Si layer, on the other hand, thermo-acoustic conversion is enhanced to a practical level. A temperature fluctuation produced at the surface of nc-Si layer is quickly transferred into air, and then an acoustic wave is emitted without any mechanical vibrations. The non-resonant and broad-band emissivity with low harmonic distortions makes it possible to use the emitter for generating audible sound under a full digital drive and reproducing complicated ultrasonic communication calls between mice.

## Introduction

As the scaling of integrated silicon devices approaches 10 nm or below, precise control of the physical and chemical properties of silicon becomes very important. In the quantum-size region of silicon (<4.7 nm), particularly, optimal processing is critical to enhance the optical, electrical, thermal, interfacial, and mechanical properties. Porous silicon (PS), prepared by electrochemical anodization of crystalline silicon (c-Si) under the certain conditions, consists of a nanopore structure and residual quantum-sized nanocrystalline silicon (nc-Si). With appropriate surface passivation, nc-Si shows tunable properties in different ways from those of bulk c-Si and plays a role as a platform of functional devices in photonics, electronics, biometrics, biomedicine, acoustics, energetics, and so on (Sailor, 2012; Canham, 2017). From among these possibilities, the studies on the emission of photons, electrons, and ultrasound are discussed here.

Regarding the photonic applications, one key issue is to develop an efficient fabrication process of highly luminescent nc-Si colloids. For this purpose, some top-down and bottom-up approaches have been conducted to obtain colloidal nc-Si dots (Heath, 1992; Henderson et al., 2009; Shirahata et al., 2010). Typical techniques in the former are laser ablation of c-Si wafer and ultrasonic fragmentation of PS. Those in the latter are chemical vapor deposition (CVD), thermal decomposition treatment, and liquid-phase chemical reaction. In any case, an energy- and cost-effective process is strongly required for producing a practical amount of nc-Si dots or powder. Although the complicated nanostructure in PS layers seems to impede the electrical conduction, on the other hand, experimental and theoretical analyses of electron transport in arrayed nc-Si dots suggests the existence of a specific multiple-tunneling cascade mode therein (Koshida, 2017a). This leads to quasiballistic electron emission from an nc-Si diode. Its usefulness has been demonstrated not only in vacuum but also in atmospheric-pressure gases and solutions. Due to extremely low thermal conductivity and volumetric heat capacity of the nc-Si layer (Lysenko et al., 1999; Valalaki and Nassiopoulou, 2013, 2014, 2017; Koshida, 2017b), in addition, thermo-acoustic coupling with air is enhanced. Since no mechanical vibrations are involved, this thermally induced sound emission shows non-resonant flat frequency response (Koshida, 2017c). The nc-Si sound source can effectively reproduce complicated ultrasonic communication calls between mice.

These functional applications are different from the pursuit of scaling merits that have been sought in usual silicon device technology. The present status of technological exploration is summarized in the following sections and some recent developments are highlighted.

## Diversifying Studies of Porous Silicon

While the minimum size of advanced large-scale integrated (LSI) circuit enters into the region below 10 nm, another viewpoint relating to environmental, social, and human issues has become important in pursuing the silicon device technology. Actually, the International Technology Roadmap for Semiconductors was recently reorganized^1^ such that some new intentionality and keywords are contained [such as systems, beyond CMOS (complementary metal-oxide-semiconductor) logic, emerging research materials, and so on] in addition to the conventional scaling activity “more Moore.” The silicon technology has reached the phase of evolutionary transformation from straightforward scaling to diversification, systematization, and functional combinations.

**Figure 1:**
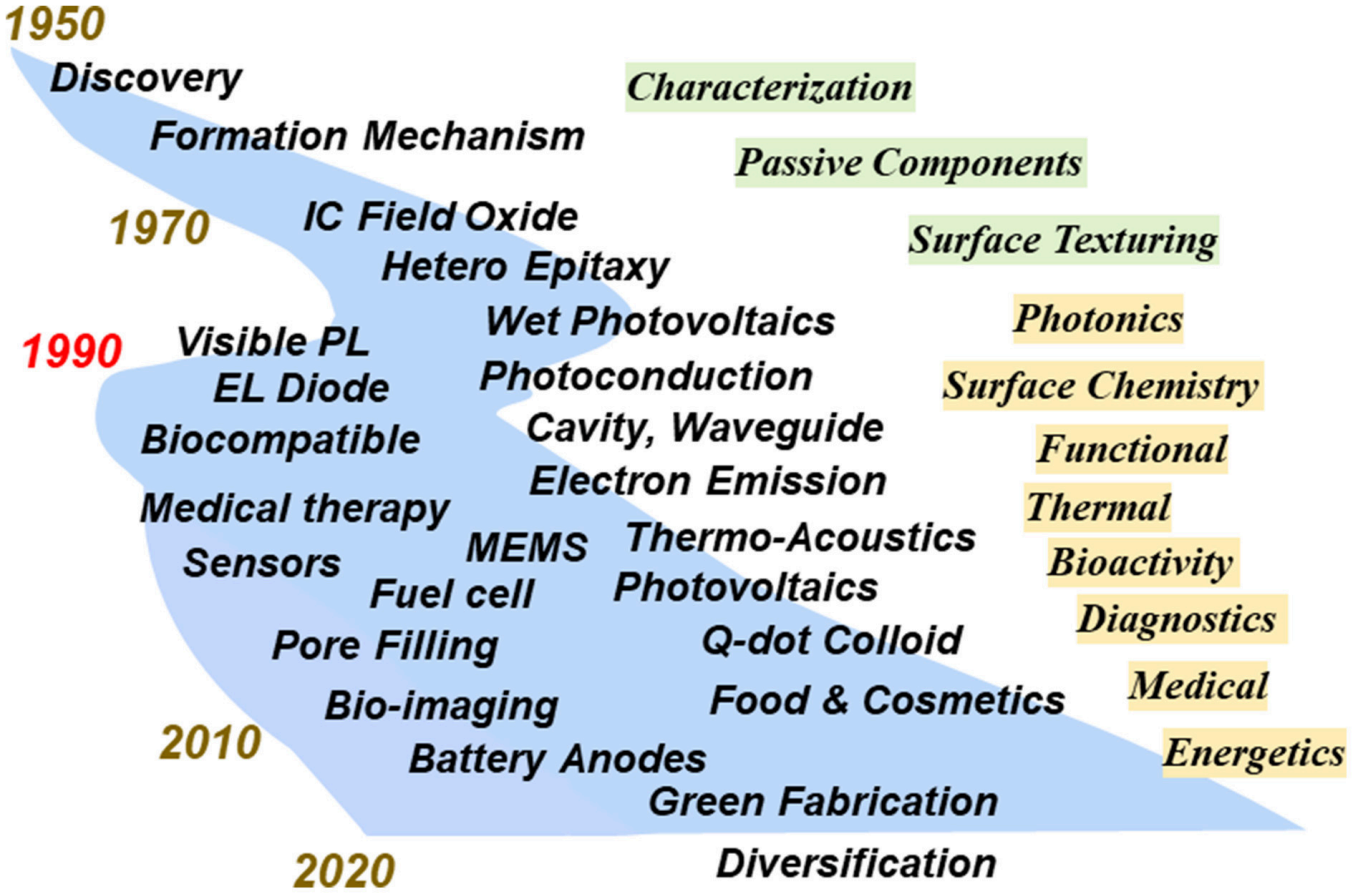
Evolution of research and development activities in nanostructured porous silicon.

The scientific and technological evolution of PS materials is shown in Figure 1. Reflecting the versatile structures of PS, its research and development (R&D) have been conducted in many divergences. In the early stage just after finding of PS by Uhlir (1956), the interests of PS were mainly in the formation mechanism and structural characterizations (Lehman, 2002). The application studies were field oxide formation for integrated device isolation (Watanabe et al., 1975) and use as substrates for epitaxial growth of compound and elemental semiconductors (Lin et al., 1987). Discovery of visible photoluminescence by Canham (1990) at 1990 led to the expansion of concern from the use as passive components to as an active quantum confinement material. Related investigations were also reported around that time on the photoelectrochemical solar cell (Koshida et al., 1985), photoconduction (Koshida et al., 1991), and electroluminescence (Koshida and Koyama, 1992), and optical effects (Thonissen et al., 1997). It was clarified that the physical and chemical properties of PS become radically different from those of single-crystalline bulk silicon. Then, the continuing studies paved the way for advanced surface chemistry (Coffinier and Boukherroub, 2016), biocompatibility (Canham, 1995), bio-sensors (Lin et al., 1997), biomedical therapy (Santos, 2014), quasiballistic electron emission (Koshida et al., 1999), thermal isolation (Nassiopoulou and Kaltsas, 2000; Nassiopoulou, 2014), and thermoacoustics (Shinoda et al., 1999). Recently the studies are further expanded to the field of energetics (Kouassi et al., 2012). Tunable optical, electrical, structural, surface, thermal, and chemical properties of PS meet the above-mentioned situation that silicon technology is rapidly evolving in a multilateral manner.

## Emissive Properties and Applications

### Visible Luminescent Quantum Dots

#### Bottom-Up Fabrication Routes of Silicon Quantum Dot

Free-standing mono-dispersed colloidal particles of nc-Si (nc-Si dots) with diameter range of ~2–8 nm is well known to exhibit size-tunable visible luminescence. Both the dispersibility of nc-Si dots in solution and luminescence properties can be controlled by modifying their surface termination (Dohnalová et al., 2014). Recently, because of such interesting properties and the resultant compatibility of future solution-based luminescence devices, such as flexible electroluminescence diode (Choi et al., 2018), the nc-Si dot attracts a lot of attention. In addition, silicon dots are expected to apply in bio-technology, such as cellular imaging, due to non-toxicity of silicon (Cheng et al., 2014). For these applications, scalable production routes of the luminescent nc-Si dots are expected to develop. In this section, we review various routes for the nc-Si dots formation and the recent advances in the efficient production of the nc-Si dots, including the processes where PS (an assembly of nc-Si dots) is utilized as an intermediate material.

**Figure 2:**
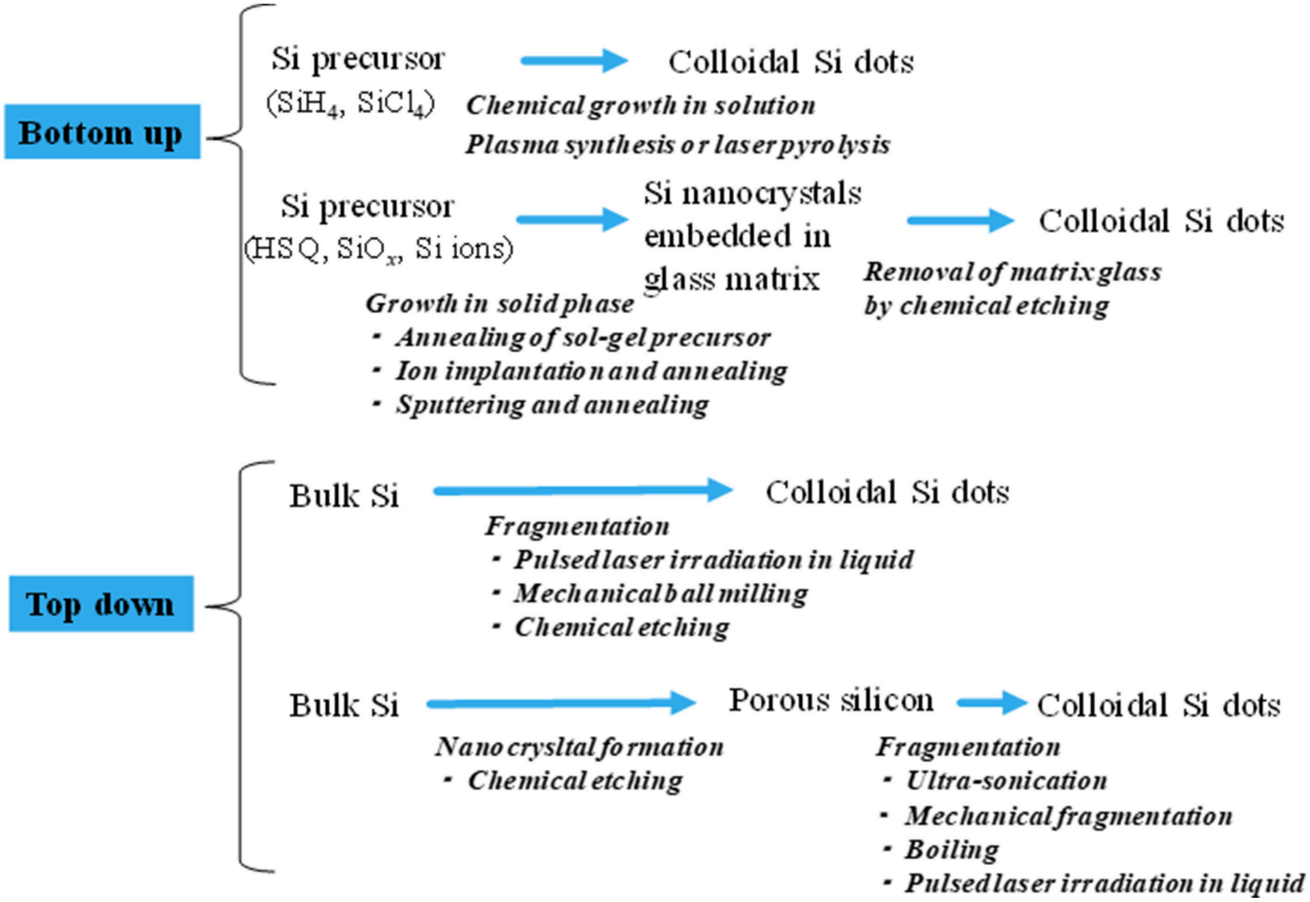
Summary of typical production processes for nc-Si dots. The bottom-up and top-down routes are shown in up and lower parts. Each process sequence represents arrows from left to right.

The nc-Si dots are prepared through the two types of preparation routes, i.e., top-down and bottom-up routes as summarized in Figure 2. A typical bottom-up process is the solution-phase chemical synthesis (Heath, 1992; Wilcoxon et al., 1999; Holmes et al., 2001; English et al., 2002; Zou et al., 2004; Liu et al., 2005; Dohnalová et al., 2012, 2013; Cheng et al., 2015; Debenedetti et al., 2015; Ghosh et al., 2018). In this method, the reduction of silicon precursors, such as SiCl_4_ with Zintl salts (KSi, NaSi, Mg_2_Si), at high temperature under high pressures forms colloidal nanocrystals. To render soluble the silicon colloids in arbitrary solvents (polar or nonpolar solvents), their surface termination usually modifies from initial termination (e.g., Br and Cl) to organic ligands. In some cases, further ligand exchanges from a ligand (e.g., alkene and thiol groups) to another ligand or biomolecules were performed for the organically-capped silicon colloids (Shiohara et al., 2010; Ruizendaal et al., 2011). The prepared colloids usually exhibit an emission in blue to green regions with nanosecond lifetimes, indicating the surface-related or direct gap recombination (Holmes et al., 2001; Dohnalová et al., 2012, 2013). Furthermore, by attaching adequate surface ligands, yellow to red luminescent colloids can be obtained. Interestingly their luminescent quantum efficiency increases up to ~90% (Qi et al., 2016). Note that the quantum efficiencies of typical nc-Si dots, where the quasi-direct electron-hole recombination occurs, were up to 60% (Jurbergs et al., 2006).

**Figure 3:**
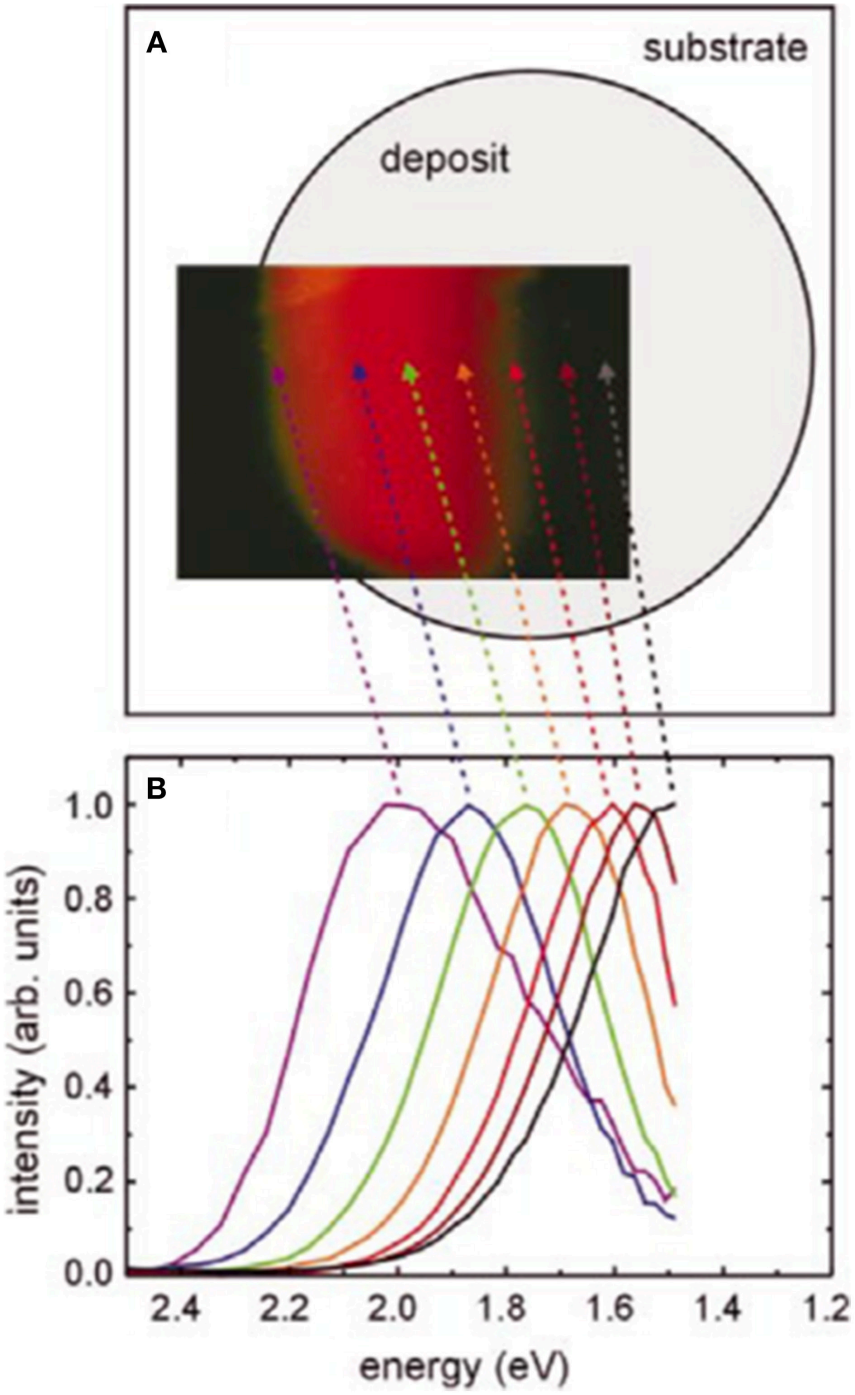
**(A)** Pictures of the size separated nc-Si dots prepared by laser pyrolysis and **(B)** corresponding PL spectra. Reprinted with permission from Ledoux et al. (2002), Copyright 2013 AIP Publishing.

Another typical bottom-up process is laser pyrolysis (Ehbrecht et al., 1997; Ehbrecht and Huisken, 1999; Ledoux et al., 2002; Li et al., 2003, 2004; Hua et al., 2006) or plasma synthesis (Mangolini et al., 2005; Nozaki et al., 2007; Anthony and Kortshagen, 2009; Gupta et al., 2009; Shen et al., 2010; Miller et al., 2012; Askari et al., 2015) from the silicon precursor such as silane. In these methods, the treatment of a pulsed laser or an induction of high frequency power leads to the decomposition of precursor molecules and formation of Si clusters. Subsequently, the growth of the silicon nanoparticles occurs due to the aggregation of the generated clusters. The obtained nanoparticles consist of the single phase crystalline core and the surrounding amorphous layer of SiO_*x*_ (Ledoux et al., 2002; Mangolini et al., 2005). Then, to adequately terminate the surfaces or further control the nanoparticle size, stain etching was performed in HF/HNO_3_ aqueous solution, where the oxidation of the silicon surface of the nanoparticles occurs and the removal of the oxide layer leads to the decrease in the size (Li et al., 2003; Gupta et al., 2009). Subsequent organic capping may also be formed by an additional chemical treatment (Li et al., 2004; Hua et al., 2006). Due to such size control processes by stain-etching, the PL emission colors of the formed nanoparticles were tuned in all visible spectral regions (Gupta et al., 2009). The emission color of the nanoparticles also changes from blue to green by the total pressure of the plasma reactor (Shen et al., 2010). In the case of the laser pyrolysis, an excellent size separation was demonstrated by using a molecular-beam chopper synchronization of the irradiation pulsed laser combining the time-of-flight mass spectroscopy (Ehbrecht et al., 1997; Ehbrecht and Huisken, 1999). Formation of such size-separated nc-Si dots reveals the clear size-dependent PL data in a red spectral region (Figure 3), and an excellent agreement between the theory and data was shown (Ledoux et al., 2002). Typical PL quantum efficiencies of the nanoparticles prepared by laser pyrolysis are 1–30% depending on their size, i.e., the larger (smaller) nanoparticles with a diameter of 8 nm (3.5 nm) have lower (higher) efficiencies (Ledoux et al., 2002).

**Figure 4:**
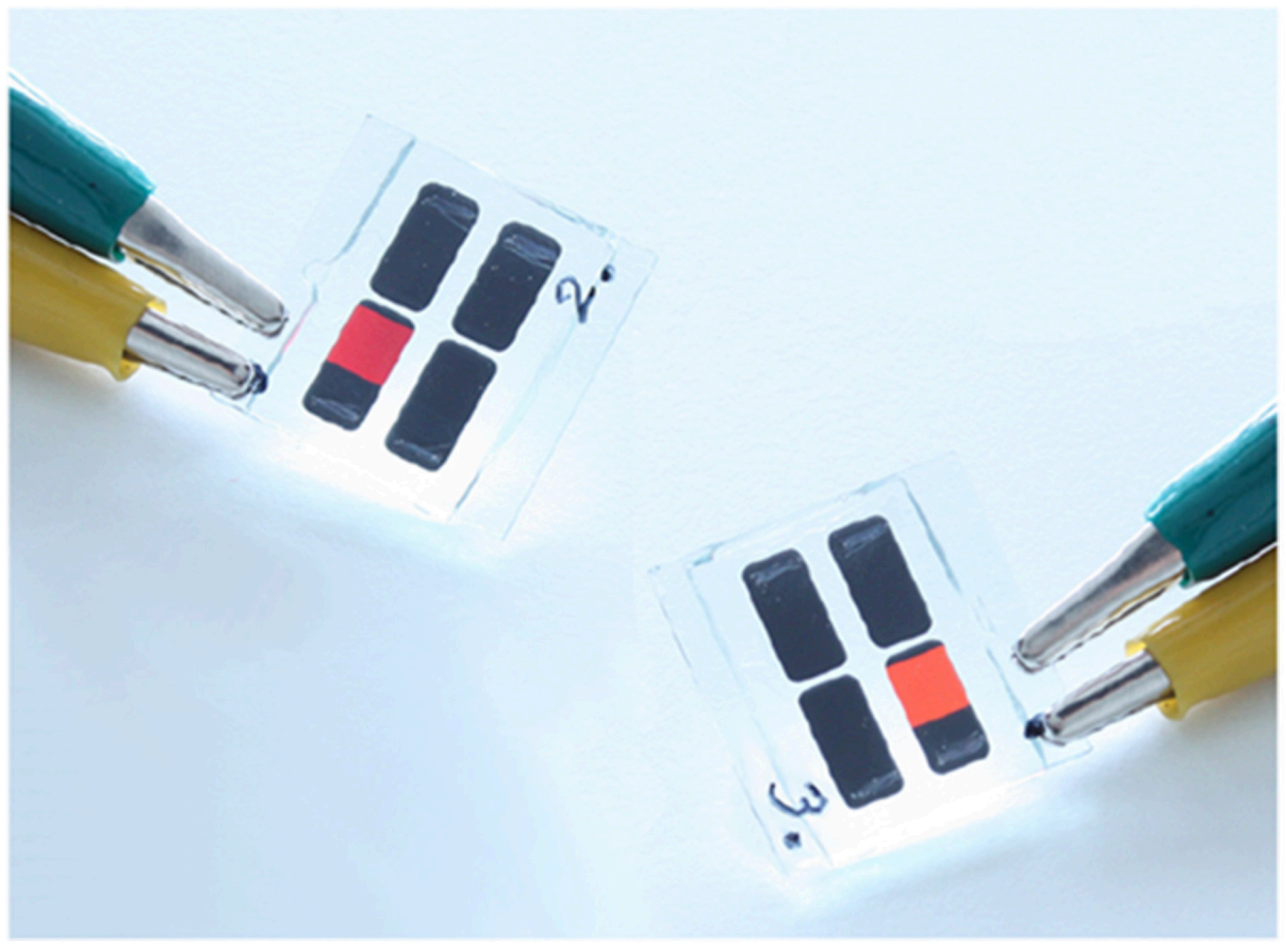
Photographs of the multicolor light emitting diodes utilizing the size-separated silicon nanocrystals prepared by the thermal process of the sol-gel precursor (hydrogen silsesquoxane). Reprinted with permission from Maier-Flaig et al. (2013), Copyright 2013 ACS Publishing.

Recently, a new type of bottom-up process using sol-gel precursors for the formation of the nc-Si dots has been developed by Veinot's Group (Hessel et al., 2006, 2008; Henderson et al., 2009; Clark et al., 2010; Kelly et al., 2010). After the annealing of hydrogen silsesquoxane (HSiO_1.5_) at high temperature (900–1400°C) under the H_2_/Ar atmosphere, nanocrystalline silicon forms in the oxide matrix (Hessel et al., 2006). With varying annealing temperature and/or annealing time, the size of the nanocrystals can be controlled. After the nanocrystal formation, HF treatment of the nanocrystal embedded oxide liberates the nanocrystals as a freestanding form due to the etched removal of the oxides, i.e., the formation of the hydrogen-terminated colloidal silicon nanoparticles. The various organic termination can be formed by the subsequent photo- or thermally-induced hydrosililation between the hydrogen surface of silicon nanoparticles and unsaturated organic species, which can make the colloidal nanoparticles soluble in desired types of solvents (Clark et al., 2010; Yu et al., 2013). The colloidal silicon nanoparticles prepared by this process exhibit usual quantum confinement induced size-dependent PL in the yellow to red region (Hessel et al., 2006). However, by attaching particular organic functional groups on the surface of the nanoparticles, the emission color can be tuned in all visible range (blue to red) without size control of dots, e.g., the diphenylamine functionalized dots shows the yellow emission (Dasog et al., 2014). These PL emission colors were independent on the polarity of the solvent and the excitation wavelength, indicating that the origin of the PL is the recombination at unknown surface states. The recombination lifetimes of such surface-related PL emission are much faster (several nanoseconds) than that of the usual quasi-direct electron-hole recombination due to quantum confinement effect. Note that their PL quantum efficiencies are 20–30% (Dasog et al., 2014). By using the silicon nanoparticles prepared by this sol-gel precursor process, solution-based multicolor light emitting diodes having high external quantum efficiencies ~1.1% were demonstrated (Figure 4) (Maier-Flaig et al., 2013) with combining the size separation technique (Mastronardi et al., 2011). Successful demonstration of such diode is thanks to the ease of the preparation handling and mass productivity as discussed below. Moreover, Ghosh et al. reported the improved sol-gel precursor process to form brighter silicon nanoparticles under mild condition, and fabricated white- (Ghosh et al., 2014) and red-emitting diodes (Ghosh et al., 2018). In addition to these sol-gel precursor process, the HF etching process of SiO_*x*_ films incorporated in crystalline silicon nanoparticles, which is prepared by radio-frequency sputtering method (Shinoda et al., 2006; Sugimoto et al., 2012) and subsequent thermal annealing, provides the formation of the colloidal nc-Si dot.

#### Top-Down Fabrication Routes of Silicon Quantum Dot

One of the most simple top-down processes is the mechanical fragmentation of bulk silicon and/or silica by a ball milling technique (Lam et al., 2000; Heintz et al., 2007). In particular, Heintz et al. (2007) demonstrated the formation of blue-emitting quantum dots with alkyl-termination by combining the high energy ball milling of a bulk silicon chunk and a simultaneous chemical reaction with unsaturated organic species. Furthermore, chemically size reducing technique by stain-etching of bulk silicon or silicon rich oxide powders have been reported (Sato and Swihart, 2006; Sato et al., 2009; Goller et al., 2010). Sato et al. demonstrated the PL color tuning (green to red) of the quantum dots (2–3 nm) by stain-etching of the polycrystalline powder with an average diameter of 50 nm in HF/HNO_3_ aqueous solution with ultrasound treatment which allows uniform etching (Sato et al., 2009). Furthermore, Goller et al. reveal that spherical quantum dots (3–10 nm) formed by stain-etching of silicon powder (25 nm), and they show a single exponential PL decay curves (Goller et al., 2010), in contrast to stretched exponential curves for usual silicon nanocrystals including PS (Pavesi and Ceschini, 1993). This is considered to be attributed to a uniform spherical shape of the dots.

Laser ablation of bulk silicon in liquid, such as water (Švrček et al., 2006; Umezu et al., 2007), organic solvent (Shirahata et al., 2010; Abderrafi et al., 2011), and supercritical fluid (Saitow and Yamamura, 2009) is a simple fabrication route for the nc-Si dots. Umezu et al. demonstrated that the irradiation of pulsed laser light (532 nm, 10 mJ/cm^2^) to a bulk silicon wafer in hexane and water generates blue-emitting oxide-capped colloidal silicon nanoparticles (Umezu et al., 2007). Švrček et al. revealed that the size of the nanoparticles depends on the irradiation laser power in the diameter range from 2 to 10 nm, and higher power laser irradiation causes the fragmentation of the aggregated nanoparticles (Švrček et al., 2016). Shirahata et al. demonstrated the pulsed laser irradiation to a bulk silicon wafer in unsaturated organic solvents such as 1-octene yields organically-capped silicon nanoparticles (Shirahata et al., 2010). The nanoparticle formation mechanism in these laser ablation methods in liquid is as follows: Intense pulsed laser light ablates bulk silicon target, resulting in the formation of the silicon vapors and/or clusters. Then, the ablated silicon vapor condensed into the silicon nanoparticles at the liquid/vapor interface. In particular, using the unsaturated organic solvent, chemical reaction between the surface of the nanoparticles and unsaturated bonds of the organic solvent causes the efficient surface termination, resulting in a relatively higher PL quantum efficiency (~10%). However, in these pulsed laser ablation methods, the formed silicon nanoparticles have relatively larger size distribution and an exact size control are essentially difficult.

**Figure 5:**
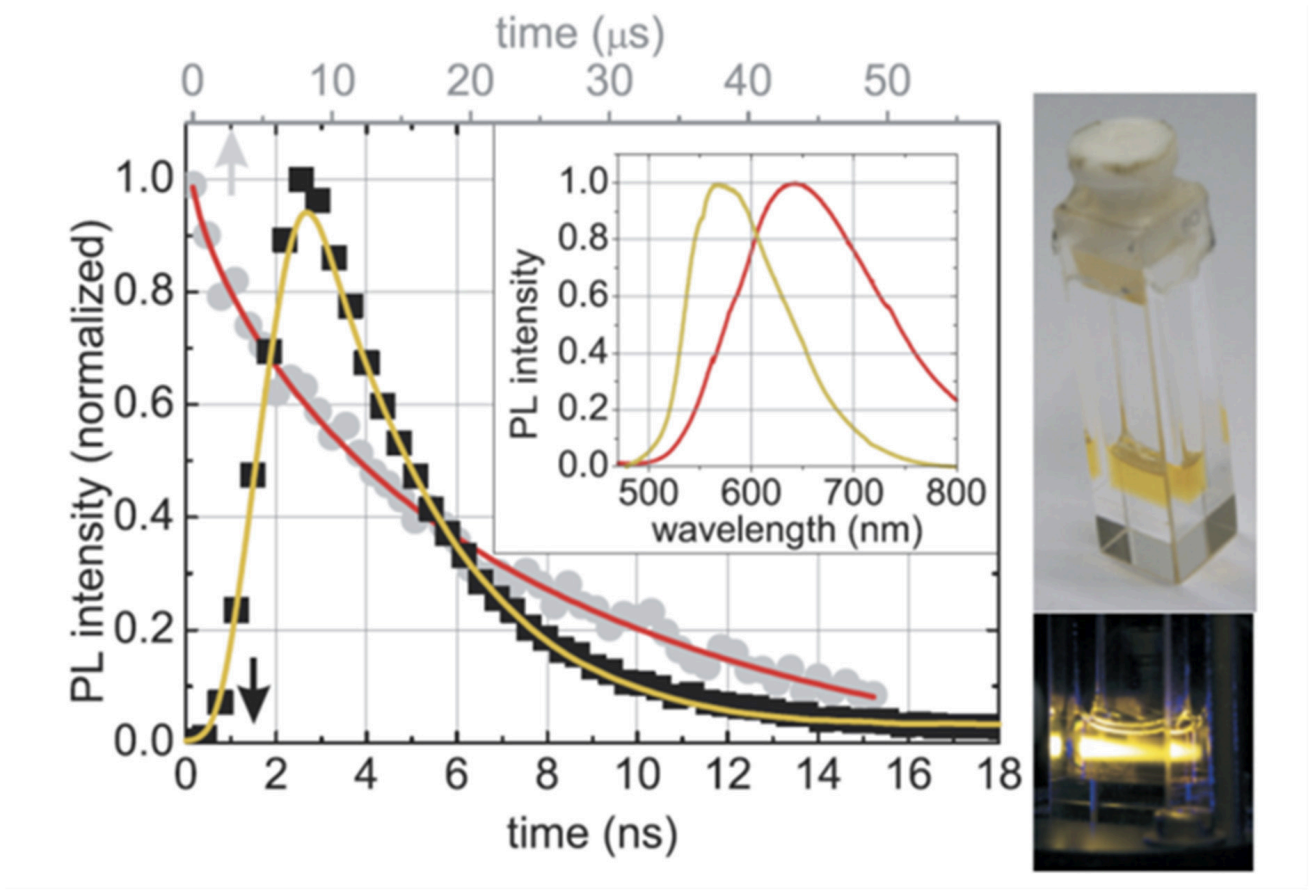
Yellow emitting nc-Si dots with nanosecond PL decay. Reprinted with permission from Kusová et al. (2010), Copyright 2010 ACS Publishing.

An important top-down process for nc-Si dots is the formation of the PS by electrochemical etching of silicon wafer and subsequent pulverization of the porous layer. As the porous layer consists of the assembly of nc-Si dots, a relatively *mild* pulverization treatment can render the porous layer free-standing nanoparticle form. Several pulverization techniques have been employed, such as ultra-sonification (Heinrich et al., 1992), and mechanical milling (Ryabchikov et al., 2012; Luna López et al., 2014). Heinrich et al. firstly demonstrated that the ultra-sonification of the PS layer in various solvents, such as methanol, toluene, and water, generates the colloidal silicon nanoparticles (Heinrich et al., 1992). However, the obtained colloidal nanoparticles have a wide size distribution from several nanometers to hundreds of nanometers. Thus, to purely obtain light emitting nanoparticles (a dimeter range from 2 to 10 nm) due to quantum confinement effect, additional size separation procedures are usually needed. For example, the subsequent filtering of the supernatant part of as-prepared colloidal solution was performed (Valenta et al., 2008). Furthermore, the additional chemical etching of the as-prepared colloidal nanoparticles was also employed to obtain colloidal samples having controllable visible PL emission from green to red (Choi et al., 2007; Kang et al., 2009). The surface of the obtained colloidal silicon nanoparticles prepared by this method are oxygen or hydrogen terminations. The silicon nanoparticles formed by the pulverization of the PS usually have the similar PL emission properties as original PS, although they exhibit a blue shift of the PL peak due to being free from matrix stress (Kusová et al., 2012) and the apparent increase in PL quantum yields (Credo et al., 1999). Organically capped nanoparticles can be also obtained by an additional chemical treatment, i.e., the photo-assisted hydrosilylation in organic solvent (Buriak, 2009). Kusová demonstrated the formation of yellow emitting organically capped nc-Si dots prepared by combining the sonification of porous layer and subsequent photo-assisted hydrosilylation treatments (Figure 5) (Kusová et al., 2010). Interestingly, these colloidal nanoparticles exhibit the nanosecond PL decay, due to electron-hole direct gap recombination induced by the crystalline strain and resultant modification of the electronic band structure (Kusová et al., 2014). Another simple process to pulverize the PS is boiling of the PS in an organic solvent with unsaturated bonding (Lie et al., 2002; Chao et al., 2007). This treatment leads to the formation of alkyl-capped silicon nanoparticles with a diameters of ~2.5 nm, resulting from the bubble formation by hydrosilylation between the unsaturated organic solvent and hydrogen-terminated silicon surface (Phatvej et al., 2018), and resultant pulverization of PS.

#### Efficient Approaches for the Silicon Quantum Dot Production

There are various criteria to evaluate the productivity for the nc-Si dot fabrication. Askari et al. summarized the figure of merit such as absolute throughput (kgh^−1^) and throughput density (kgh^−1^m^−2^), of above mentioned various processes (Askari et al., 2015). In this subsection, we summarize recent advances in the view of the production quantity per experimental batch. Zhong et al. produced ~10 g of green emitting green-quantum dots from ~100 g silicon precursor (C_6_H_17_NO_3_Si) by solution-phase chemical process for 30 min (Zhong et al., 2015). Here, we define the production yield as the ratio of the weight of the produced quantum dots to that of original silicon material. The production yield per batch of Zhong's process is ~10%. In the high annealing process of sol-gel precursor (~2 h per batch), typically 20 mg of the hydrogen terminated nc-Si dots were produced from 200 mg of HSQ silicon precursor, corresponding to the production yield of ~10% (Islam et al., 2017). Bose et al. demonstrated that the quantum dots can be prepared from the reduction of rice husk by rapid microwave heating (Bose et al., 2018). The production yield of this process is ~5%, i.e., 0.1 g of nanoparticles from 2 g of rice husk per batch. Note that the plasma synthesis from silane precursor provides ~20 mg quantum dots per batch (~45 min) with almost 100% of production yield, representing the complete conversion from the silane precursor (Mangolini et al., 2005). We summarize the production yields for these processes in Table 1.

**Table 1:** Typical production yields of nc-Si dot per batch in various processes.

	**Bottom-up process**			**Top-down process**	
	**Sol-gel precursor thermal process (Islam et al., 2017)**	**Chemical synthesis (Zhong et al., 2015)**	**Plasma synthesis (Mangolini et al., 2005)**	**Reduction by microwave thermal process (Bose et al., 2018)**	**Improved pulsed laser irradiation (Nakamura et al., 2018)**
Si precursor	HSQ	C_6_H_17_NO_3_Si	Silane	Rice husk	PS
Nanocrystal formation process	High temperature annealing	Chemical reduction growth	Plasma decomposition	Thermal reduction	Chemical etching
Liberation process	Chemical etching	None	None	Milling and sedimentation	Pulsed laser irradiation
Amount of precursor [mg]	200	10^4^	200	2000	10
Amount of quantum dots [mg]	20	10^3^	200	100	8.5
Production yield [%]	10	10	100	5	85

**Figure 6:**
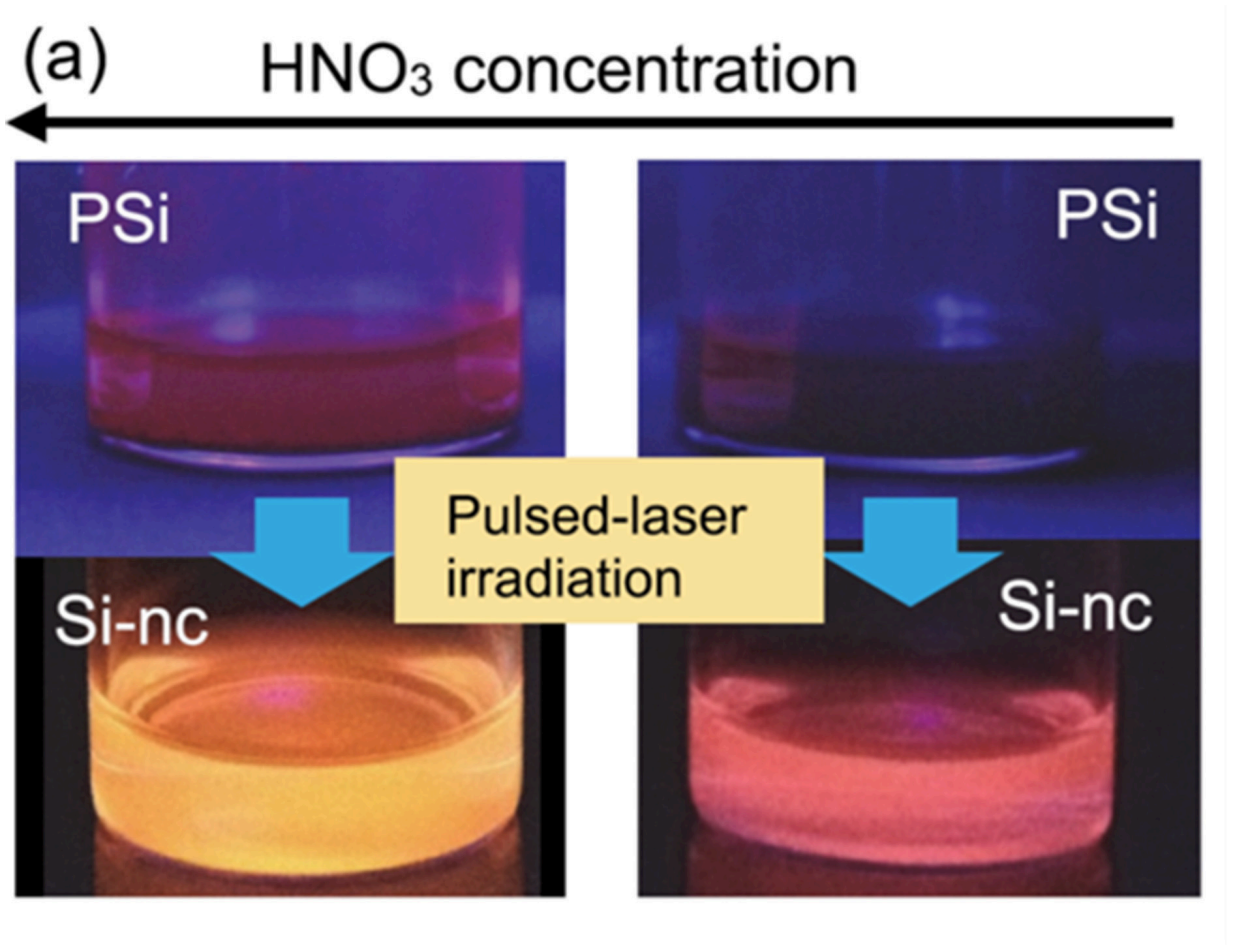
Pictures of multicolor PL emission from nc-Si dots produced by the pulsed laser irradiation to PS prepared by different etching conditions. Reprinted with permission from Nakamura et al. (2016), Copyright 2016 AIP Publishing.

Very recently, Nakamura et al. demonstrated that the pulsed laser irradiation of the PS powder in organic solution efficiently generates the nc-Si dots and the formed quantum dots exhibited the multicolor PL emission in blue (Nakamura et al., 2014), white (Yuan et al., 2017a), and red (Nakamura et al., 2016; Yuan et al., 2017b) regions. The PL quantum efficiencies are much higher (~10–30%) than the original PS (~1%) (Nakamura et al., 2016). The prepared quantity of the quantum dots is more than ten times larger than the usual pulsed laser ablation method using the bulk silicon target (Nakamura et al., 2014). The formation mechanisms depend on laser irradiation conditions, i.e., the ablation of porous layer and subsequent condensation into nanoparticles, or the pulverization of the porous layer resulting from the laser induced thermal stress. In the case of the pulverization induced formation of the dots, the PL emission color was able to be controlled via etching condition of the target PS (Figure 6) (Nakamura et al., 2016), i.e., the changes in the size of nanocrystalline porous network core. As described in the above subsection, such PL emission color control was usually difficult in the usual laser ablation process using bulk silicon target. This efficient fabrication of the nc-Si dots is attributed to unique thermal properties of PS. The PS has a much smaller thermal conductivity (in the range of 0.5–1.0 W/mK), which is comparative to insulators such as quartz glass and rubber (Lysenko et al., 1999; Valalaki and Nassiopoulou, 2013, 2014, 2017; Koshida, 2017b). In addition to the conductivity, heat capacities are also very low (0.2–0.6 MJ/m^3^K) in contrast to the insulators (Koshida, 2017b). These unique thermal properties of PS cause a local heating inside the porous layer when the pulse laser irradiated to it, and the efficient ablation or fragmentation occurs. By improved pulsed laser irradiation process for 10 mg of PS, ~8.5 mg of red emitting nc-Si dots has been produced (Nakamura et al., 2018). This production amount of the quantum dot per batch is much larger than the boiling process of PS, i.e., several hundred micro grams of dots from 1 cm^2^ of silicon chip wafer (Dickinson et al., 2008; Alsharif et al., 2009). Moreover, the production yield of this process (~85%) is larger than the above mentioned chemical synthesis and high temperature annealing process of sol-gel precursor (Islam et al., 2017) (see Table 1). Thus, the demonstrated laser induced heating process of PS provides an energy- and cost-effective route for production of nc-Si dots.

### Quasiballistic Electron Emission

#### Emission Mechanism and Characteristics

**Figure 7:**
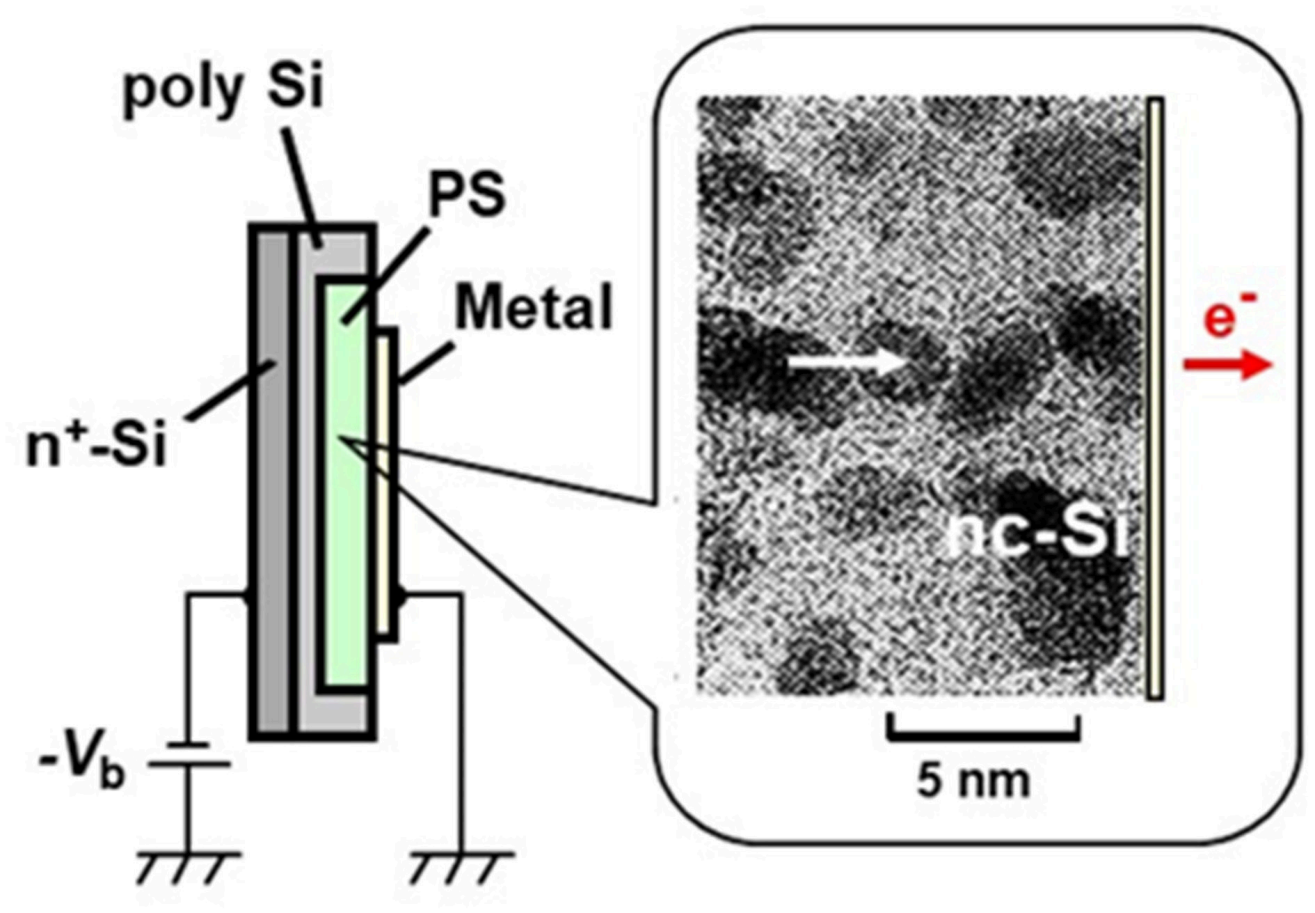
Schematic structure of PS-based quasiballistic electron emitter. A cross-sectional transmission electron microscopy (TEM) photograph of PS layer is also shown.

The device is composed of a thin film surface electrode, a PS layer (~1 μm thick), a silicon wafer substrate, and a back contact (Figure 7). As observed by transmission electron micrograph (TEM) shown in this figure, the PS layer prepared by galvano-static anodization and additional oxidation includes nc-Si dots (~3 nm in mean diameter) interconnected with tunnel oxides. In this PS layer, there is a multiple-tunneling transport mode through nc-Si dot chain, and quasiballistic hot electrons are efficiently generated (Mori et al., 2011). Under the condition that a positive voltage is applied to the surface electrode with respect to the substrate, electrons are accelerated in the PS layer toward the outer surface, and then some of them are emitted through the surface electrode. The emission starts at an onset voltage corresponding to the work function potential of the surface electrode. The applied voltage dependence of the emission current follows the Fowler-Nordheim tunneling scheme. The emission efficiency η, defined as the ratio of the emission current density to the diode current density, depends on the nanostructure arrangement of nc-Si dots, quality of interfacial tunneling oxide, and the surface electrode material.

When a monolayer graphene is used as a surface electrode, the typical η value is drastically enhanced to 6.3% at an applied voltage of 10 V (Kojima et al., 2018a) due to a high transparency of monolayer graphene for quasiballistic electrons. At that applied voltage, the mean energy of output electrons becomes higher than 2 eV. The corresponding electron temperature is far from the thermal equilibrium. The mean energy of emitted electrons can be tuned well by the applied voltage while keeping narrow energy dispersion. Both the output electron energy distribution and the emission angle dispersion become significantly narrow even at room temperature. The energy distribution becomes more monochromatic at a low temperature of around 150 K. The measured emission angle dispersion is just ±8° with respect to the surface normal (Kojima et al., 2018b). The relatively low operation voltages and the compatibility with silicon planar processing make it possible to drive the emitter array under an active-matrix mode.

#### Applications of Quasiballistic Electron Source

##### (i) Availability for varied media

Being the energetic, directional, planar, and uniform emission, the quasiballistic emission from PS is insensitive to vacuum pressure, in contrast to the conventional cold cathodes such as field emitters and metal-insulator-metal ones. Far from it, the PS emitter operates in gases and even in solutions. The application studies have been carried out in vacuum (flat panel display, multibeam parallel lithography, high-sensitivity image sensor), in atmospheric pressure gases (negative ion generation, non-discharge VUV emission), and in solutions (H_2_ gas evolution, pH control, thin film deposition) (Koshida, 2017a). As specific approaches, two topics on the development that demonstrates the characteristic feature of the PS emitter are presented here.

##### (ii) Multibeam parallel lithography

In advanced silicon device technology, a high resolution (below 10 nm), high throughput, and cost-effective nanofabrication process is strongly required. Though electron beam (EB) is a very attractive exposure source from a viewpoint of the resolution, the conventional focused EB writer has a seriously limited throughput. If a practical multibeam exposure scheme could be possible, the usefulness of mask-less EB direct-write should be dramatically enhanced. Its major possible applications are photomask fabrication and mask-less direct-write exposure. Specifications of multibeam parallel lithography systems under development are summarized in Table 2.

**Table 2:** Developing studies of multibeam parallel EB lithography.

**Group**	**EB Source**	**Mode**	**Demagnification**	**Beam**	**Voltage (kV)**	**Use**	**Reference**
IMS^a^	Thermionic	Aperture blanking	1/200	512 × 512	50	Photo-mask writer	Klein et al., 2012; Platzgummer et al., 2013; Klein and Platzgummer, 2016
NuFlare^b^			1/200	512 × 512	50		Matsumoto et al., 2016
MAPPER^c^			1/1	13,260	5	Direct-write	Rio et al., 2010; Brandt et al., 2015
TU^d^ and TUAT^e^	nc-Si ballistic emitter	Active-matrix drive	1/1000	100 × 100	5		Esashi et al., 2015

a IMS Nanofabrication AG, Austria;

b NuFlare Technology, Japan;

c MAPPER Lithography, The Netherlands

d Tohoku Univ., Japan;

e *Tokyo Univ. of Agri. and Tech., Japan*.

**Figure 8:**
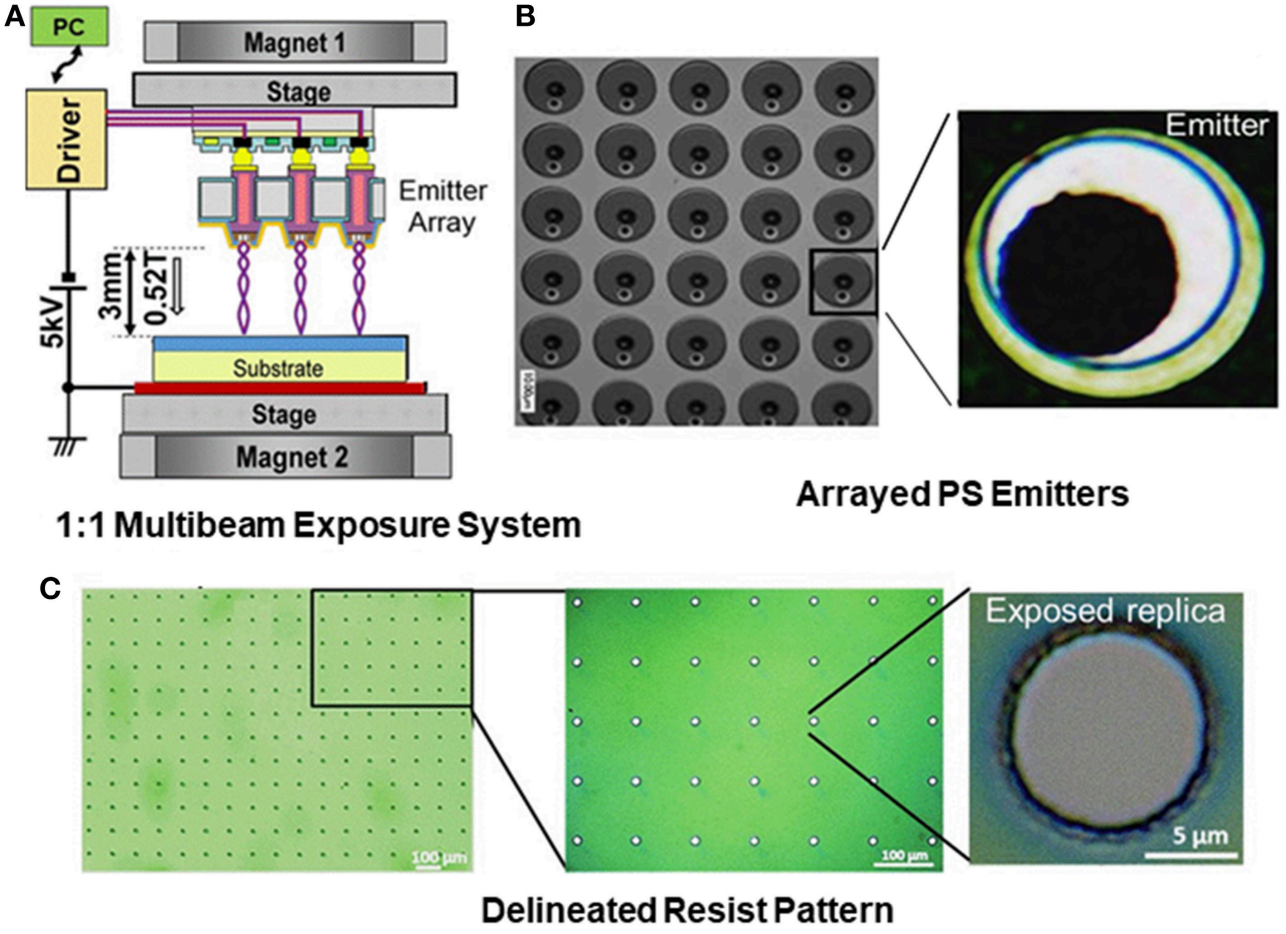
**(A)** Schematic configuration of 1:1 electron multibeam exposure system using arrayed nanocrystalline PS emitters. **(B)** SEM photographs of emitter array. The emission area is 12 μm in diameter in this case. **(C)** Delineated resist patterns.

In the conventional systems, thermionic emitter or thermally assisted field emitter is used as an electron source. Since the employment of active-matrix drive is difficult in that case, broadened electron beam is spatially switched by aperture blanking method for generating multibeam (Rio et al., 2010; Klein et al., 2012; Platzgummer et al., 2013; Brandt et al., 2015; Klein and Platzgummer, 2016; Matsumoto et al., 2016). In contrast, the PS approach is characterized by active-matrix drive of arrayed emitters (Esashi et al., 2015). The PS emitter array can be fabricated on a Si-wafer substrate by planar processes. The back contact of each electron emitter with an active area of 10 × 10 μm^2^ is interconnected to an active matrix driving circuit using a through-silicon-via (TSV) technique (Figure 8A). A CMOS-based LSI circuit has been developed for the multibeam (100 × 100) parallel lithography.

The compatibility of the implemented LSI with the active-matrix operation was confirmed, including the basic function for the electron emitter process variation compensation and the test of integrated devices. The evaluation was performed with the 1:1 exposure test system, in which an EB-resist coated target wafer was placed at about 3 mm distance from the emitter surface (Figure 8B) and the exposed resist pattern is shown in Figure 8C. It has been demonstrated that the integrated nc-Si emitter array is compatible with the active-matrix drive for multi-beam massive parallel exposure, and that the selected emitter pattern is delineated corresponding to the activated emitters. In accordance with the results of beam optics simulation in the prototype system, the miniaturized electron optics is suitable for 10 nm order EB writing. For the practical use with a throughput comparable to extreme ultra-violet (EUV) lithography, criteria of the electron beam number and the resolution target to be pursued are 10^6^ beams and 5 nm, respectively.

##### (iii) Reductive deposition of thin films

**Figure 9:**
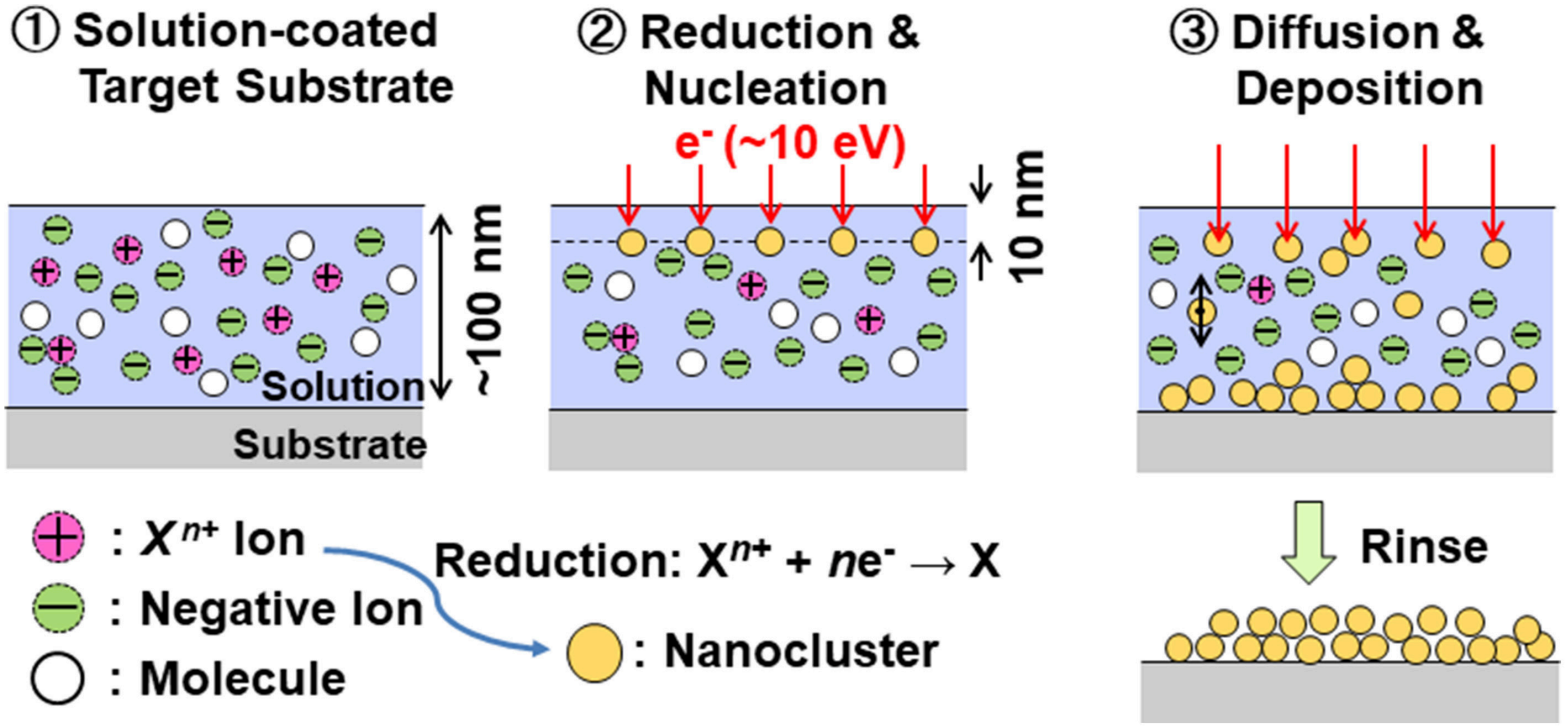
Process of liquid-phase reductive thin-film deposition promoted by direct incidence of quasiballistic electrons emitted from nanocrystalline PS cold cathode.

From a chemical viewpoint, the PS emitter can be regarded as a supplier of electrons with highly reducing activity. Its direct application is liquid-phase thin film deposition of metals and semiconductors under an electron incident mode (Suda et al., 2016). The deposition process is illustrated in Figure 9. Output of quasiballistic electrons of the nc-Si emitter impinges onto the target substrate on which an extremely small amount of salt solutions such as CuCl_2_, SiCl_4_, and GeCl_4_ was coated in advance with a thickness of 100 nm. The spacing between the emitter and substrate was controlled in the range from 500 nm to 100 μm by a piezoelectric actuator, taking the relation between the electron mean free path and the vacuum pressure of used solution into account. The experiments were done in a N_2_-gas filled glove box.

**Figure 10:**
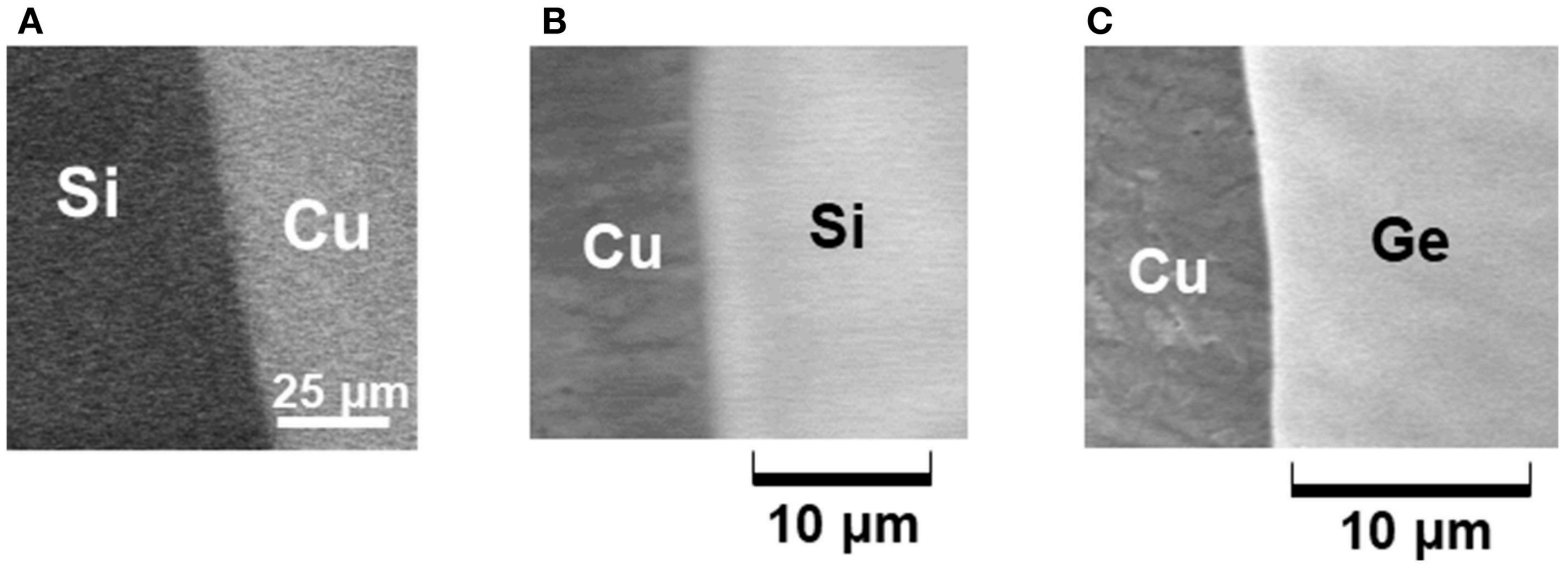
SEM photographs of deposited thin films of Cu **(A)**, Si **(B)**, and Ge **(C)**.

After the emitter operation for a few minutes, residual solutions were removed, and then thin Cu, Si, and Ge films are formed on the incident area as shown in Figure 10. According to the structure and compositional characterizations of deposited thin films, every film consists of nanoclusters. No contaminations were detected by X-ray photoelectron spectroscopy (XPS). Obviously thin films are deposited with no byproducts. Thin films can be deposited at room temperature on varied substrates including insulating layers (i.e., oxidized c-Si wafer) and flexible polymers. In addition, a mixture solution such as SiCl_4_+GeCl_4_ is available for deposition of thin SiGe films with a controllable composition.

Incident electrons with energy of 10 eV can penetrate 10 nm deep in solutions (Emfietzoglou et al., 2009), and reduce positive ions therein followed by the formation of nanoclusters and deposition. Thermodynamic investigation supports that the incident electron energy meets the requirement for preferential nucleation of atoms rather than their out-diffusion (Suda et al., 2017). The theoretical analysis based on the reaction diffusion equation suggests that the deposition rate depends mainly on the incident electron current density *J*_e_, and that it reaches a stationary value within 0.1 s after electron incidence (Suda et al., 2018). At the typical condition of *J*_e_ = 10–100 μA/cm^2^, the estimated stationary deposition rate of Cu, Si, and Ge films are around 0.2–2.0 nm/min. This is consistent with the experimental results.

Typical thin film deposition techniques are summarized in Table 3. The most widely used dry processes (chemical and physical vapor deposition) are established by precise control of temperature, vacuum pressure, and gas flow rate (Seshan, 2012). The wet electroplating, based on exchange of thermalized electrons at the working and counter electrodes, proceeds at room temperature with gas evolutions. It is mainly used to deposit thin metal films (Schlesinger and Paunovic, 2010). Electron-beam-induced deposition (EBID), on the other hand, has been studied to form cluster, metal nanowires, thin films, and nanostructures (Kiyohara et al., 2002; Adelung et al., 2004; Gazzadi and Frabboni, 2005; van Dorp et al., 2005; Randolph et al., 2006; Frabboni et al., 2008; Furuya, 2008; van Dorp and Hagen, 2008; Botman et al., 2009; de Boer et al., 2011; Vollnhals et al., 2013; den Heijer et al., 2014; Leenheer et al., 2015). The focused electron beam with high-energies of 10–50 keV in the conventional scanning or transmission electron microscope is transmitted through membranes and then hits the absorbed gases or ionic liquids on the substrate leading to decomposition of molecules. The key issue is to reduce carbon and other contaminations in deposited thin films. The ballistic electron incidence mode mentioned above is based on the mechanism different from EBID. Unilateral reduction proceeds with neither gas evolution nor by-product generation. In addition, the deposition of thin metal and group IV semiconductor films is available for varied substrates.

**Table 3:** Comparative survey of thin film deposition processes.

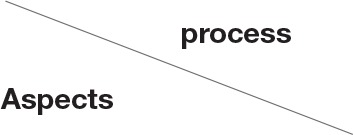	**Dry process (CVD^a^ and PVD^b^)**	**Electroplating**	**Electron Irradiation**	
			**EBID^c^ (10**~**50 keV)**	**Ballistic incidence (**~**10 eV)**
Phase	Vapor or vacuum	Liquid	Liquid or vapor	Liquid
Mode	Decomposition, sputtering, or evaporation	Redox reaction	Decomposition	Reduction
Temperature	High	Room temperature		
Contamination	<ppb	Gas evolution	C, O	<300 ppm

a *Chemical Vapor Deposition*,

b *Physical Vapor Deposition*,

c *Electron Beam Induced Deposition*.

### Thermo-Acoustic Emission and Applications

The PS acoustic devices are composed of a thin-film surface heater electrode, a PS layer, and a c-Si wafer. Due to a strong phonon confinement and interfacial scattering in PS, the thermal conductivity of PS layers, α, is drastically lowered in comparison to that of bulk silicon (Lysenko et al., 1999; Valalaki and Nassiopoulou, 2013, 2014, 2017; Koshida, 2017b). At the same time, its volumetric heat capacity *C* is also significantly decreased. In the case of high-porosity PS, particularly, both α and *C* values become close to the lower limit of solid state materials. Thus, the thermal diffusivity $\sqrt{\alpha C}$ is extremely decreased. When a temperature fluctuation is produced by electrical input to the heater electrode, a significant acoustic wave is generated near the surface, because the thermos-acoustic transfer effect is inversely proportional to $\sqrt{\alpha C}$ (Shinoda et al., 1999). A significant sound pressure amplitude is produced without any mechanical vibrations.

Due to the sound emission from still surface, the frequency response covering a fully wide range is free from the mechanical resonance. The theoretical limit of frequency response is 1 GHz. No resonant peaks are observed in the whole range of available frequency. The broad-band flat emissivity of the PS device is useful for reproducing complicated ultrasonic communication calls and male-female interactions between mice (Kihara et al., 2006; Uematsu et al., 2007). Conventional ultrasound emitters cannot be utilized for this application because of a resonant frequency response and a bulky size larger than mice. As previously demonstrated, mouse mothers were attracted by pup ultrasonic vocalizations (USVs) reproduced by an nc-Si emitter, while they did not respond to other synthesized sounds. It was also found that the response to pup USVs was enhanced by social experiences (Okabe et al., 2013). Recent study on mutual recognition between mother and infant suggests that pup USVs looks to have an individual signature used in pup differentiation by mouse mothers, similar to acoustic communication between human mothers and their infants (Asaba et al., 2015; Mogi et al., 2017).

Regarding thin metal film heaters and underlying thermal insulators, many studies have been conducted by using varied combinations: suspended Al wires-air (Niskanen et al., 2009), Si nanowires-polymer or -glass (Tian et al., 2011a), indium-tin-oxide film-glass (Daschewski et al., 2015), Si nanoparticles-sapphire (Odagawa et al., 2010), conducting polymers-glass (Tian et al., 2011b), thin Au film-porous polymer (Chitnis et al., 2012), thin Ag–Pd film-glass-Al_2_O_3_ (Nishioka et al., 2015), carbon nanotube (CNT)-air (Xiao et al., 2011), or -grooved Si (Wei et al., 2013), graphene-polymer (Suk et al., 2012; Tian et al., 2014; Kim et al., 2016; Tao et al., 2016; Sbrockey et al., 2018), -porous Al_2_O_3_ (Tian et al., 2012), or -glass (Fei et al., 2015), CNT-laser-scribed graphene-polymer (Yeklangi et al., 2018), and W-Al_2_O_3_-polymer (Brown et al., 2016). The basic characteristics of these devices are consistent with the theoretical analyses of the thermo-acoustic effect and its key factors (Hu et al., 2010, 2012a,b, 2014; Vesterinen et al., 2010; Daschewski et al., 2013; Lim et al., 2013; Yang and Liu, 2013; Wang et al., 2015; Tong et al., 2017; Xing et al., 2017). Making use of the non-resonant and broad-band emissivity with no harmonic distortions, possible applications have been pursued to audible compact speaker under a full digital drive, probing source for 3-dimentional object sensing in air, acoustic pressure generator for noncontact actuation, directivity control under phased array configuration, loud speaker, noise cancellation, thermoacoustic tomography, and thermoacoustic sound projector (Koshida, 2017c; Aliev et al., 2018; Bobinger et al., 2018; Julius et al., 2018; Liu et al., 2018; Song et al., 2018).

## Summary

Including photonic visible luminescence, emerging functions of nanostructured PS has extended to electronics, biometrics, biomedicine, acoustics, thermology, and energetics. In the quantum-size silicon, especially, the emissive properties of photons, electrons, and sound are activated. From a technological viewpoint, cost- and power-effective production of luminescent nc-Si powder or colloid is desired for wide applications. As one practical approach, high-yield fabrication of strongly luminescent colloidal nc-Si dots has been developed by employing *in-situ* self-regulated process for pulverization of anodized PS by pulsed laser irradiation. A multiplier tunneling transport through nc-Si dots, on the other hand, induces quasiballistic electron emission. The potential of nc-Si cold cathode has been made clear by using monolayer graphene as a surface electrode. This makes the foundation more solid for applications to massively parallel EB lithography under an active-matrix drive and to reductive thin film deposition of metals and semiconductors. Based on specific thermal properties of PS, on the other hand, thermos-acoustic device has been developed. Observed broad-band non-resonant sound emission from a compact PS device provides standard ultrasound source for researches in the bio-acoustic communications. These studies meet in the direction and requirements for diversification of silicon technology.

## Author Contributions

NK: overview, ballistic electron emission, thermo-acoustic device. TN: visible luminescent Si quantum dots.

### Conflict of Interest Statement

The authors declare that the research was conducted in the absence of any commercial or financial relationships that could be construed as a potential conflict of interest.

## Footnotes

^1^IRDS. *IEEE International Roadmap for Devices and Systems (IRDS)*. Available online at: https://irds.ieee.org
